# A Case of Primary Aldosteronism Masquerading as Bartter and Gitelman Syndromes

**DOI:** 10.7759/cureus.75644

**Published:** 2024-12-13

**Authors:** Narasingolu M Hasini, Atul K Gupta, Akash Priyadarshi, Ahmad Alam, Saif Quaiser

**Affiliations:** 1 Department of Medicine, Jawaharlal Nehru Medical College and Hospital, Aligarh Muslim University, Aligarh, IND; 2 Rajeev Gandhi Centre for Diabetes and Endocrinology, Jawaharlal Nehru Medical College and Hospital, Aligarh Muslim University, Aligarh, IND

**Keywords:** cacna1h, case report, doxycycline, drug-induced hypokalemia, familial hyperaldosteronism, primary aldosteronism

## Abstract

Primary aldosteronism (PA) is a common cause of secondary hypertension, with familial hyperaldosteronism (FH) contributing to a lesser number of cases. FH type IV, a rare subtype, has hardly been reported as a subtype of PA cases. We present a case of a 27-year-old female who presented to the emergency department with circumoral tingling and numbness. A diagnosis of hypocalcemia due to vitamin D deficiency was made. During hospital stay, she developed acute gastroenteritis and was treated with doxycycline, after which she experienced persistent hypokalemia. Further investigation revealed urinary potassium loss and metabolic alkalosis, although her blood pressure remained normal throughout her stay. Clinical exome sequencing identified a mutated variant in the calcium voltage-gated channel subunit alpha1 H (CACNA1H) gene associated with FH type IV. Elevated plasma aldosterone and suppressed renin confirmed PA. The administration of doxycycline for treating acute gastroenteritis likely precipitated hypokalemia by enhancing the expression of the mutated CACNA1H* *gene variant, thereby increasing aldosterone production.

## Introduction

Primary aldosteronism (PA) was originally described by Litynski in 1953 and later by Conn in 1954. It is now recognized as the most common cause of secondary hypertension, contributing significantly to cardiovascular and renal morbidity and mortality [1]. PA can cause a variety of symptoms, including high blood pressure, low potassium levels, fatigue, excessive thirst, frequent urination, headache, muscle cramps, and weakness. PA encompasses both sporadic and genetic subtypes. The sporadic forms primarily include unilateral aldosterone-producing adenoma (APA) and bilateral adrenal hyperplasia [2,3]. Genetic variants of PA include familial hyperaldosteronism (FH) types I-IV, FH type I is caused by CYP11B1/CYP11B2 (cytochrome P450 family 11 subfamily B members 1 and 2) chimeric gene mutation, FH type II due to CLCN2 (chloride voltage-gated channel 2) pathogenic variants, FH type III due to KCNJ5 (potassium inwardly rectifying channel subfamily J member 5 gene) pathogenic variants and FH type IV due to CACNA1H (calcium voltage-gated channel subunit alpha1 H) pathogenic variants. One more entity, earlier known as FH type V, is there which, along with PA, is associated with seizures and neurological abnormalities (PASNA) syndrome, a non-familial genetic variant of PA which is due to CACNA1D (calcium voltage-gated channel subunit alpha1 D) pathologic variants. Familial forms account for less than 10% of all PA cases [4,5]. Type I FH is also known as glucocorticoid-remediable aldosteronism (GRA). FH-II is the most prevalent subtype, whereas FH type IV is exceedingly rare, whose actual prevalence is not known, but one study was found to have FH type IV representing less than 0.1% of PA cases [4]. The CACNA1H gene plays a critical role in FH type IV by encoding the alpha-1 subunit of the T-type calcium channel Cav3.2. This channel is primarily expressed in the adrenal zona glomerulosa, where it regulates aldosterone production. In FH-IV, specific mutations in the CACNA1H gene lead to a gain-of-function effect, causing the Cav3.2 channel to be more active, and this increased activity results in elevated intracellular calcium levels in the adrenal zona glomerulosa cells, as shown in the following figure in the next section.

Genetic testing plays a crucial role in the diagnosis and management of PA and its familial forms. It helps in confirming the diagnosis of PA, identifying its familial forms, and differentiating between GRA, i.e., FH-I and non-GRA. This differentiation is important for tailoring treatment strategies. Genetic testing also helps in screening the family members of the individuals with FH to identify those at risk of developing the condition.

PA is traditionally characterized by hypertension and hypokalemia resulting from renin-independent hypersecretion of aldosterone by either a nodule or a hyperplastic adrenal cortex in the zona glomerulosa of the adrenal glands. However, recent estimates suggest that only 9-37% of patients with PA exhibit hypokalemia [6]. In rare instances, patients with PA may present without hypertension and thus can masquerade as Bartter and Gitelman syndromes when they have concomitant hypokalemia [7]. Bartter and Gitelman syndromes are rare genetic disorders that affect the kidney's ability to reabsorb sodium and chloride, which leads to excessive salt diuresis, causing electrolyte imbalance, including potassium and magnesium. Bartter syndrome is a more severe form with symptoms including muscle weakness, cramping, and even growth delays. Gitelman syndrome, on the other hand, is generally milder, having symptoms like muscle cramps, fatigue, and sometimes renal stones. Patients with PA are more likely to have hypovitaminosis D than people with essential hypertension or non-PA patients. Here, we present a case of PA manifesting as hypokalemia with normal blood pressure, which, upon evaluation and clinical exome sequencing, revealed heterozygous CACNA1H mutation, was diagnosed as PA due to FH type IV.

## Case presentation

The clinical data are being presented after obtaining an informed written consent from the patient herself. A 27-year-old primigravida female with six months of gestation presented to the emergency department with circumoral tingling and numbness, difficulty in swallowing, and opisthotonos for the first time in her life. There was also no history of any recent wounds, injuries, or punctures that could suggest tetanus. There was also no history of any drug intake or similar complaints in the past, and the family history was also insignificant. On physical examination, her blood pressure was 108/74 mmHg, which remained around the same during her hospital stay, and she had lockjaw accompanied by stiffness of muscles of the neck and back along with that of all extremities. During the initial days of her hospital admission, she also developed multiple episodes of loose stools, which resulted in the preterm delivery of her baby. She was treated with 300 mg of doxycycline stat dose, metronidazole, and crystalloids for loose stools for a total of five days, as per our institutional protocol for acute gastroenteritis. Her initial workup revealed a very low ionized calcium level, which came out to be because of the underlying vitamin D deficiency (Table 1). However, the serum potassium level at presentation was normal (4.0 mmol/L). Upon correcting the hypocalcemia, her serum potassium levels started declining two days later, which was persistent and refractory despite adequate correction.

**Table 1:** Diagnostic tests conducted for the evaluation of hypocalcemia ALP, alkaline phosphatase; iPTH, intact parathyroid hormone

Parameter (units)	Value	Reference range
Serum calcium (mmol/L)	1.5	2.12-2.62
Serum albumin (g/L)	36	35-50
Ionized calcium (mmol/L)	0.4	1.16-1.31
Serum phosphate (mmol/L)	0.9	0.8-1.4
ALP (U/L)	612	30-120
Serum creatinine (µmol/L)	70.7	53-97.2
25(OH)vitamin D (nmol/L)	11.2	75-250
iPTH (pmol/L)	18.4	1.6-6.9
Serum magnesium (mmol/L)	0.72	0.65-1.05

Investigations

The patient was subsequently evaluated for hypokalemia. Her urinary potassium excretion was >15 mmol/day, and the trans tubular potassium gradient (TTKG) was >4, indicating distal renal potassium loss (Table 2). Her blood pH was also measured, showing metabolic alkalosis, while urinary chloride levels were >20 mmol/L. These findings further guided the differential diagnosis, as shown in Table 2.

**Table 2:** Diagnostic tests conducted for evaluation of hypokalemia pH, potential of hydrogen; TTKG, trans tubular potassium gradient

Parameter	Value	Reference range
Serum potassium (mmol/L)	1.9	3.5-5
Urine potassium (mmol/day)	22.3	5-15
TTKG	8	2-4
pH	7.53	7.35-7.40
Serum bicarbonate (mmol/L)	32	16-24
Urine chloride (mmol/L)	57	20-40
Plasma aldosterone (pmol/L)	665.7	139-499
Plasma renin activity (ng/mL/hour)	0.2	1-4

Differential diagnosis

Hypokalemia can be due to increased entry into cells, gastrointestinal loss, or urinary loss. The cause of hypokalemia is usually apparent from the history (vomiting, diarrhea, diuretics). However, the cause is uncertain sometimes. The major component of the diagnostic evaluation is an assessment of urinary potassium excretion and the assessment of acid-base status. In our patient, urinary potassium excretion and TTKG suggested urinary potassium loss. The differential diagnosis of hypokalemia due to urinary loss (TTKG > 4) includes diuretics, increased mineralocorticoid activity, renal tubular acidosis (RTA), and Liddle, Bartter, and Gitelman syndromes. Our patient had metabolic alkalosis and no history of diuretic usage, which narrowed the differential to increased mineralocorticoid activity and Liddle, Bartter, and Gitelman syndromes. Lastly, normal blood pressure favored the likely diagnosis of Bartter and Gitelman syndromes. However, the normal serum potassium level at presentation was atypical for these genetic syndromes. This raised the possibility that an additional factor may have triggered the hypokalemia. To further clarify the diagnosis, a genetic study was performed.

Treatment

The patient was started on intravenous potassium supplementation till it reached 3.1 mmol/L and then was shifted to oral supplementation (total potassium supplement, 480 mmol over 72 hours). Spironolactone, a mineralocorticoid receptor antagonist at 50 mg daily as per the institutional protocol, was added to the treatment regimen. She was also advised to take elemental calcium at 1.5 g/day in three divided doses and cholecalciferol 60,000 IU weekly for 10 weeks.

Outcome and follow-up

Clinical exome sequencing revealed a heterozygous variant of uncertain significance in the CACNA1H gene at the 3' acceptor splice site in intron 33 (c.5888-3C>A). This variant affects the position of three nucleotides upstream of exon 34. Since the 3' acceptor splice site variant in the CACNA1H gene (c.5888-3C>A; ENST00000348261.11) is present right next to the exonic region and was hence sequenced and detected on clinical exome sequencing, whose primary targets are usually exome sequencing in the coding regions. The variant has not been previously reported in major databases like 1000 Genomes, gnomAD (V3.1), gnomAD (V2), and Topmed, and in silico predictions by MutationTaster2 suggest that the variant is likely deleterious (Table 3).

**Table 3:** Variant details of the CACNA1H mutation found in the patient +, positive for mutation; #, the transcript used for clinical reporting represents the canonical transcript, which is usually the longest coding transcript with strong supporting evidence; $, genetic test results are reported based on the recommendations of American College of Medical Genetics and Genomics; OMIM, Online Mendelian Inheritance in Man; PM2, allele frequency classification used in variant classification

Gene^# ^(Transcript)	Location	Variant	Zygosity	Disease (OMIM)	Inheritance	Classification^$^
CACNA1H (+) (ENST00000348261.11)	Intron 33 (chromosome 16: g.1218967C>A; depth 181x)	c.5888-3C>A (3’ acceptor site)	Heterozygous	Familial hyperaldosteronism, type IV (OMIM#617027)	Autosomal dominant	Uncertain Significance (PM2)

**Figure 1:**
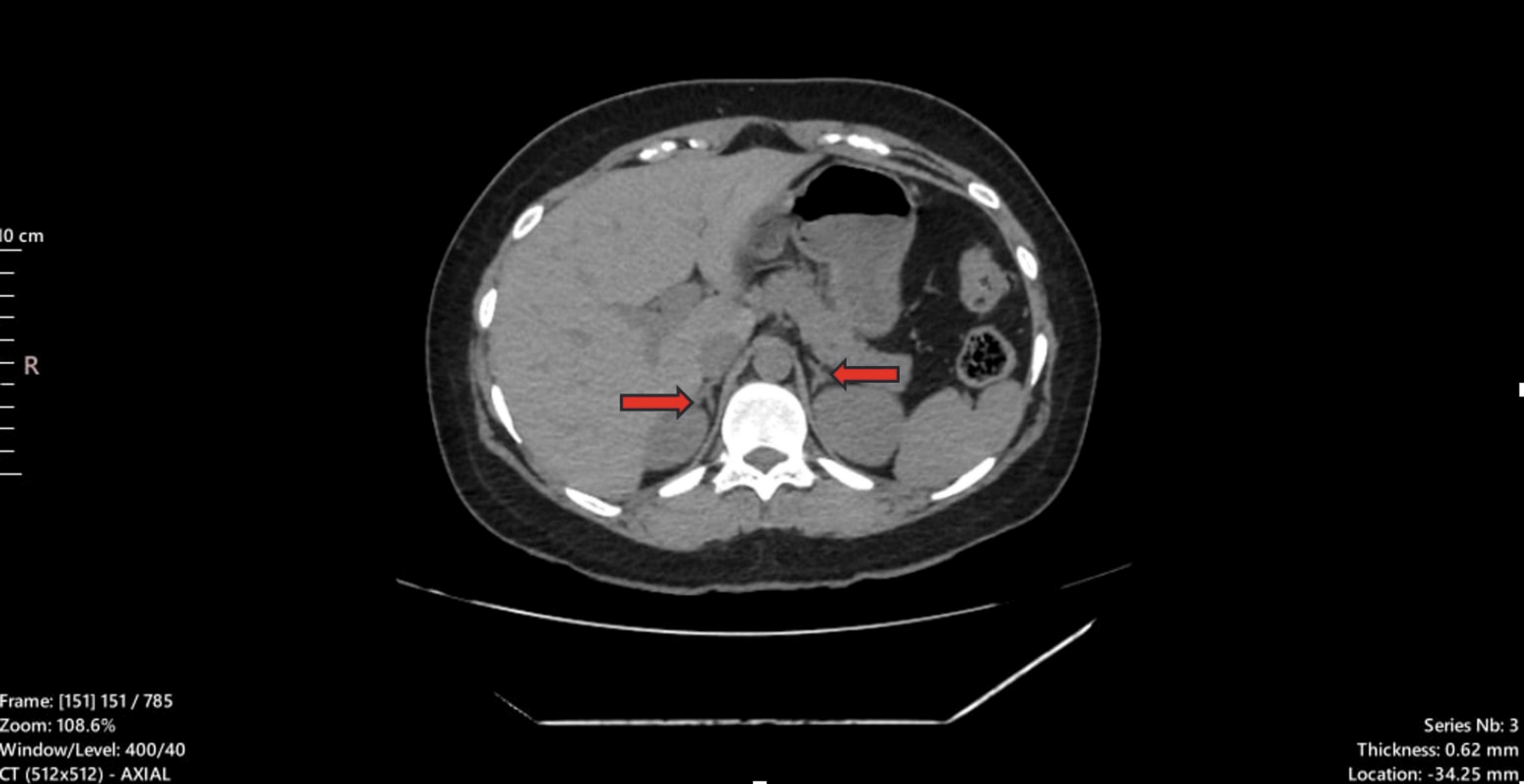
CT scan of the abdomen of the patient (axial view) showing no radiographic abnormality in the adrenal glands bilaterally, as marked by red-colored arrows. Note: The image was evaluated by a professional radiologist.

Based on these genetic findings, plasma renin and aldosterone levels were subsequently analyzed to investigate the suspected diagnosis of FH type IV. Stored plasma samples, preserved at -20°C, revealed markedly elevated plasma aldosterone levels at 665.7 pmol/L (reference range: 139-499) and suppressed plasma renin activity (PRA) at 0.2 ng/mL/hour (reference range: 1-4 ng/mL/hour). This is to note that spironolactone was stopped prior to getting the blood samples for PRA and aldosterone levels. These findings were consistent with PA associated with FH type IV (Table 2). Following this, a CT scan of the abdomen was obtained to look for any adrenal nodule, hyperplasia, or mass, but both adrenal glands were normal, as shown in Figure 1.

**Figure 2:**
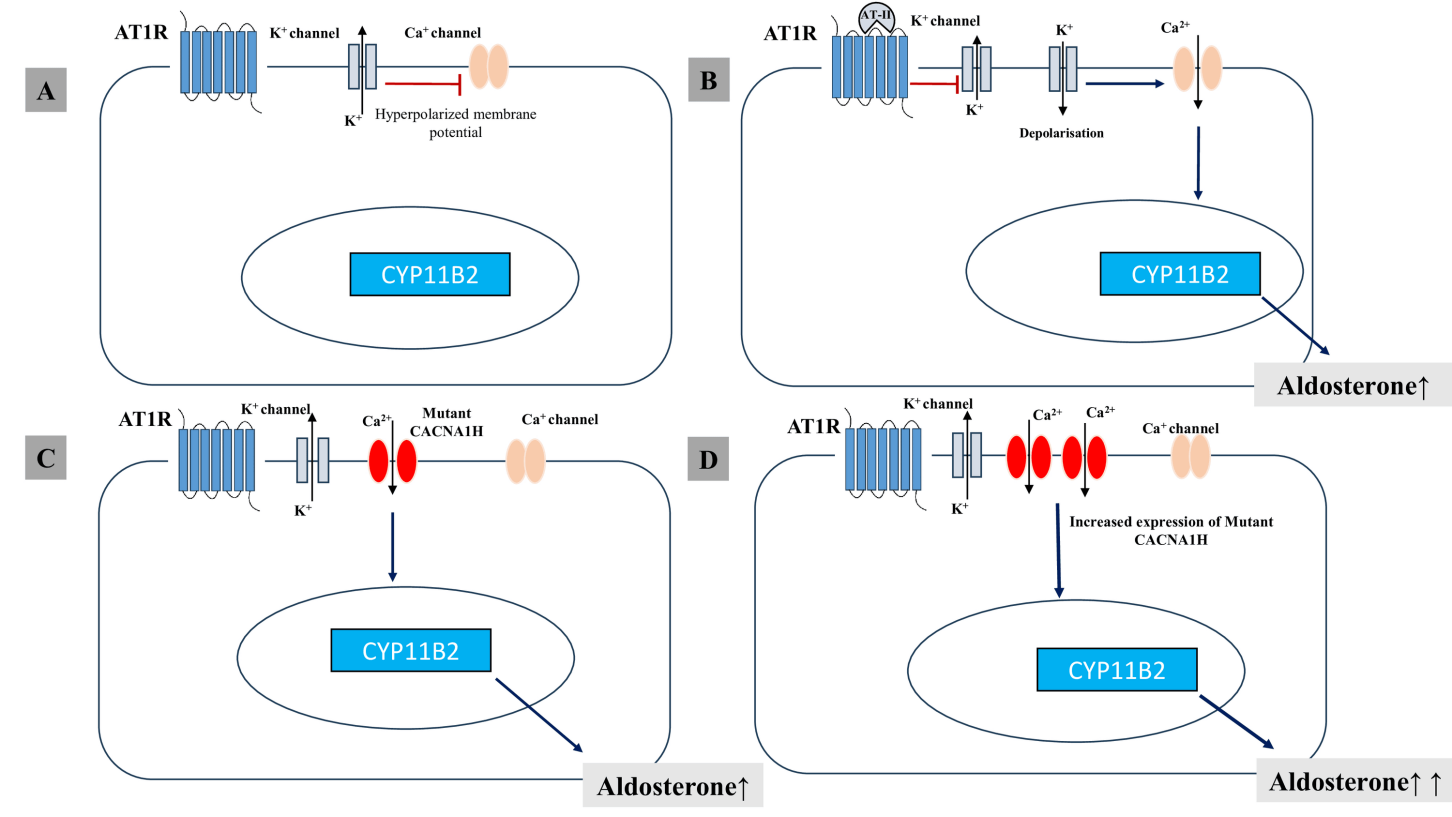
A) Under resting conditions, cells are hyperpolarized. B) Angiotension-II binds to the AT-1 receptor, causing depolarization, the opening of voltage-gated calcium channels, increased expression of CYP11B2, and increased aldosterone production. C) In familial hyperaldosteronism type 4, the presence of a mutant CACNA1H gene leads to increased calcium influx through the T-type calcium channels. D) Doxycycline further increases the expression of the mutant CACNA1H gene, leading to an amplified calcium influx, which in turn causes even greater CYP11B2 expression and aldosterone production. AT1R, angiotensin-1 receptor; AT-II, angiotensin-II Note: Figure created by the authors using Microsoft PowerPoint (Microsoft® Corp., Redmond, WA).

In our patient, the administration of doxycycline for the management of acute gastroenteritis likely precipitated hypokalemia by interacting with the pathogenic CACNA1H gene variant, potentially influencing aldosterone production, which was also demonstrated by Nanba et al., as in Figure 2 [8].

Her serum potassium level was normal during follow-up visits, and spironolactone was stopped. She remained normotensive, and her further follow-up course was uneventful. She was asked to refrain from taking doxycycline to prevent the precipitation of similar events in the future. Imaging of the adrenal glands revealed no abnormalities in bilateral adrenal glands. None of the family members had a history of hypertension or hypokalemia.

Table 4 provides a comparative overview of the clinical presentation and laboratory findings in Bartter syndrome, Gitelman syndrome, and our case.

**Table 4:** Comparison of clinical and laboratory parameters in Bartter syndrome, Gitelman syndrome, and our case "↑" = increased; "↓" = decreased; "→" = normal or unchanged; CACNA1H, gene encoding calcium voltage-gated channel subunit alpha1 H; ClC-Kb, chloride channel Kb gene that encodes kidney-specific chloride channel ClC-Kb expressed in TAL; CT, collecting duct; DCT, distal convoluted tubule; NCCT, gene encoding sodium chloride co-transporter; NKCC2, gene encoding for sodium potassium chloride co-transporter 2; TAL, thick ascending limb of the loop of Henle

Parameter	Bartter	Gitelman	Our case
Age of presentation	Early childhood	Late childhood or adolescent	Adulthood
Blood pressure	↓ or →	↓ or →	→
Hypokalemia	Yes	Yes	Yes
Urinary potassium	↑	↑	↑
Metabolic alkalosis	Yes	Yes	Yes
Hypomagnesemia	↓ or →	↓	→
Urinary chloride	↑	↑	↑
Plasma renin activity	↑	↑	↓
Plasma aldosterone activity	↑	↑	↑
Development retardation	Present	Absent	Absent
Location of K^+ ^loss	TAL	DCT	CT
Gene mutation	ClC-Kb, NKCC2	NCCT	CACNA1H

## Discussion

Since Conn's original description of APA in 1954, several subtypes of PA have been identified [1]. The most common causes include bilateral idiopathic hyperaldosteronism (IHA), or idiopathic hyperplasia, accounting for 60% to 70% of cases, and unilateral APAs, which comprise 30% to 40% of cases [9]. Less common forms include unilateral hyperplasia, characterized by micronodular or macronodular hyperplasia of the zona glomerulosa in one adrenal gland, presenting similarly to APAs, and FH, a rare inherited form with various subtypes. Classic presenting signs of PA are hypertension and hypokalemia, but potassium levels are frequently normal in the modern-day series of PA [6]. The blood pressure in PA is often substantially elevated [10]. Patients with FH present with variable degrees of hypertension, hypokalemia, metabolic alkalosis, suppressed renin, and elevated aldosterone production, typically with onset early in life. FH type IV is caused by a gain of function mutation in the CACNA1H gene, which encodes a voltage-dependent T-type calcium channel (Cav3.2), causing an increased concentration of intracellular calcium, resulting in increased aldosterone production (Figure 2) [11,12].

PA is now viewed as a continuous and evolving syndrome that can manifest across a spectrum of blood pressure phenotypes, from preclinical stages to severe hypertension [9]. In 1972, Brooks et al. first described a case of normotensive PA; since then, a hundred such cases have been reported in the literature [13]. Vantyghem et al., in 1999, reported two normotensive PA cases in middle-aged women due to APA. Both presented with hypokalemia, elevated plasma aldosterone, and undetectable renin. Post-surgery, potassium levels normalized, and both patients experienced unexpectedly low blood pressure, indicating that aldosterone excess might cause relative hypertension in those with naturally low baseline blood pressure [7]. Additionally, a study by Ito et al. in 2011 revealed that PA is not uncommon in prehypertensive and stage 1 hypertensive populations, with a prevalence of at least 6.8% in prehypertensive patients and about 3.3% in stage 1 hypertensive patients [14]. This study emphasizes the broader spectrum of PA, indicating that normotensive presentations might be more common than previously recognized, particularly in certain populations like middle-aged women. The absence of hypertension in these patients could be due to factors such as early-stage disease, reduced sensitivity to aldosterone at the vascular level, enhanced activity of vasodilators such as bradykinin, prostacyclin, and nitric oxide, or hormonal influences like estrogen, which is increasingly produced and secreted in blood by the growing placenta, can modulate blood pressure by promoting vasodilation [15-18]. Also, progesterone, whose level progressively increases during pregnancy, acts as a competitor of aldosterone in the distal convoluted tubule (DCT), thus blocking the mineralocorticoid receptors.

The diagnosis of PA in normotensive patients requires a high index of suspicion. The standard diagnostic approach, which often hinges on the presence of hypertension, may overlook these patients. Thus, clinicians should consider screening for PA in patients with unexplained hypokalemia, especially if they exhibit other features of mineralocorticoid excess.

While not a hallmark feature of PA, mild hypomagnesemia can sometimes occur due to increased renal magnesium excretion caused by aldosterone excess. However, severe hypomagnesemia is very rare in PA and is more common in conditions like Bartter and Gitelman syndromes. In PA, the high levels of aldosterone suppress the renin-angiotensin-aldosterone system, leading to low PRA levels.

Nanba et al. used a doxycycline-inducible system specifically designed to control the expression of a mutant CACNA1H gene in an adrenal cortical cell line (HAC15). This system, part of the Tet-On/Tet-Off mechanism, is an artificial tool in molecular biology that allows researchers to regulate gene expression by adding or removing doxycycline [8]. 

This setup enables the precise control of when and how much of the mutant gene is expressed, providing insights into the gene’s effects under induced conditions. Importantly, this inducible vector system does not imply that doxycycline would naturally activate the FH type IV mutation in a clinical context, as doxycycline’s effect was on the vector system used for gene regulation, not directly on the CACNA1H gene itself.

While our case suggests a possible interaction between doxycycline and the CACNA1H gene variant, this hypothesis requires further investigation in a physiological setting. Thus, caution is advised when prescribing tetracycline antibiotics in patients with known CACNA1H variants, but further research is needed to clarify the clinical relevance of this interaction.

The variant identified (c.5888-3C>A) is currently classified as a variant of uncertain significance according to the genetic report (Table 3). Its impact on protein function and potential to alter mRNA splicing remains unconfirmed. Demonstrating altered splicing and confirming a gain-of-function effect would require further validation through RNA analysis and functional studies, which we could not perform due to financial constraints. Consequently, while in silico predictions suggest this variant may be deleterious, direct evidence of its pathogenicity is currently lacking.

In our patient, the administration of doxycycline for treating acute gastroenteritis likely precipitated hypokalemia by enhancing the expression of the mutated CACNA1H gene variant, thereby increasing aldosterone production (Figure 2) [8,19]. This highlights the importance of careful consideration when prescribing tetracycline antibiotics in patients with suspected or confirmed FH type IV. The patient's follow-up showed normalization of potassium levels, and the spironolactone was subsequently discontinued, further supporting the link between doxycycline and hypokalemia in patients with FH type IV.

Learning points/take-home messages

PA can present as hypokalemia with normal blood pressure. In patients with hypokalemia and metabolic alkalosis, particularly those with low or normal blood pressure, PA should be included in the differential diagnosis alongside Bartter and Gitelman syndromes. Doxycycline can precipitate hypokalemia by enhancing the expression of the pathogenic CACNA1H gene variant in patients with FH type IV.

## Conclusions

PA, once considered a rare cause of hypertension, is now recognized as a more prevalent and clinically diverse disorder. While classic presentations involve hypertension and hypokalemia, normotensive forms are increasingly being identified, particularly in middle-aged women. The recent case report highlights the potential role of doxycycline in exacerbating hypokalemia in patients with FH type IV, emphasizing the importance of judicious antibiotic use and vigilant monitoring in individuals with a genetic predisposition to mineralocorticoid excess. Clinicians should maintain a high index of suspicion for PA, especially in patients with unexplained hypokalemia, even in the absence of hypertension.
